# Inebriate Asylum

**Published:** 1872-02

**Authors:** 


### INEBRIATE ASYLUM.

At the last meeting of the Georgia Medical Association, Dr. G. W. Holmes, of Rome, read a memorial, to be presented to the Legislature, from a number of clergymen, asking aid in the establishment of an Inebriate Asylum. The memorial was referred to that body for its indorsement, and, after its reference to a committee, the objects of the memorial were fully indorsed by the Association.

We have seen nothing in the reported proceedings of the Legislature of this memorial. We hope that the gentlemen who were so thoroughly impressed with the necessity of such an institution in our State have not grown lukewarm in the good work, but will, at the meeting of the Legislature in July, present such an array of facts as will induce that body to give the necessary aid to commence at once an institution so important to an unfortunate class of our population. The cost and working of an institution of this character would by no means be so great as many suppose. After once equipped and under way, such asylums in other States are generally self-sustaining. From the report of the Superintendent and Physician of the New York State Inebriate Asylum for 1871, we see that the receipts are two thousand dollars more than the expenditures, and that, too, with forty-one per cent. of non-paying patients.

